# Combinatorial evaluation of phase formation and magnetic properties of FeMnCoCrAl high entropy alloy thin film library

**DOI:** 10.1038/s41598-019-44351-8

**Published:** 2019-05-27

**Authors:** A. Marshal, K. G. Pradeep, D. Music, L. Wang, O. Petracic, J. M. Schneider

**Affiliations:** 1 Materials Chemistry, RWTH Aachen University, Kopernikusstr. 10, 52074 Aachen, Germany; 2 Jülich Centre for Neutron Science JCNS and Peter Grünberg Institute PGI, JARA-FIT, Forschungszentrum Jülich GmbH, 52425 Jülich, Germany

**Keywords:** Materials science, Physics

## Abstract

We report on the influence of the Al content (from 3.5 to 54 at.%) on phase formation and magnetic properties in FeMnCoCrAl high entropy alloy thin film libraries. Al additions to FeMnCoCr crystallizing in the alpha-Mn structure cause the formation of the body centered cubic (BCC) structure. This is consistent with density functional theory predictions as Al additions give rise to a larger stability for the BCC phase compared to the face centered cubic phase (FCC) which can be rationalized by the formation of a pseudogap at the Fermi level indicating the stabilization of the BCC phase over the FCC phase. Al additions to paramagnetic FeMnCoCr induce ferromagnetism. The largest saturation magnetization was measured for the film containing 8 at.% of Al. As the concentration of non-ferromagnetic Al is increased beyond 8 at.%, the number density of the ferromagnetic species is decreased causing a concomitant decrease in magnetization. This trend is consistent with ab initio predictions of the Al concentration induced changes in the magnetic moment. Based on the experimental and theoretical results presented here the effect of the Al concentration on the phase formation and the magnetic properties of FeMnCoCrAl thin film library can be rationalized.

## Introduction

Multi principal element high entropy alloys (HEA)^1,2^ have attracted a lot of attention because of their unusual properties^3,4^. Reports suggest that the mechanical performance of some of these alloys^3,4^ surpasses the performance of conventional alloys, for instance, combined increase in strength and ductility^3^ and improved mechanical properties at cryogenic temperatures^4^. These alloys are composed of multiple elements with equiatomic or near equiatomic concentrations exhibiting different crystal structures and magnetic properties^1^. In this work we adhere to the HEA definition of Yeh *et al*.: HEAs are “composed of five or more principal elements^1^” and “HEAs may contain principal elements with the concentration of each element being between 35 and 5 at.%^1^”. According to Yeh *et al*.^1^ a large negative mixing entropy in a multicomponent metallic system lowers the tendency for ordering and/or segregation, thereby promoting the formation of random solid solutions. Nonetheless, several studies revealed that the configurational entropy is not the only criterion determining phase formation and that the equiatomic alloys are not necessarily characterized by superior properties^5,6^. Also the formation of phases in addition to a single solid solution phase has been observed^6,7^. Recently, non-equiatomic HEAs, where the concentration of one or more of its constituents are varied to tailor the properties, have attained significant interest^3,5,8,9^. In fact, they even outperform some of their equiatomic variants, regarding mechanical properties^3,9^. The equiatomic FeMnCrCo HEA exhibits a multiphase microstructure with brittle Cr rich sigma phase as one of its phase constituent^9^. Whereas, the non-equiatomic Fe_40_Mn_40_Cr_10_Co_10_ HEA forms a single FCC phase with high tensile strength and elongation owing to twinning induced plasticity at room temperatures^9^. Likewise, the superior strength-ductility combination of the non-equiatomic Fe_50_Mn_30_Cr_10_Co_10_ is attributed to the transformation induced plasticity and microstructural design^10^.

With HEAs at the brink of application^4^, most of the reported work on HEAs were focused towards microstructure and mechanical behavior^3,4^. Limited studies on functional (including magnetic) properties of HEAs have been reported^11–15^. For example Lucas *et al*. described a tunable magnetic transition temperature as a function of Pd content in the FeNiCoCrPd HEA system^12^, making them suitable for magnetic refrigeration applications. FCC FeCoNi(AlSi|)_x_ based HEAs were reported to exhibit saturation magnetization (M_s_) values as large as 1.15 T at room temperature^14^. However, FeNiCoCr and FeMnCoCrNi FCC HEA systems exhibit room temperature paramagnetism^12^, while Al_x_CoCrFeNi^11^ as well as Al_x_CrCuFeNi_2_^16^ are ferromagnetic at room temperature. It was reported that the presence of a secondary ordered Al-Ni phase in AlCoCrFeNi decreases the overall M_s_^11^ and that Al additions to CoCrFeNi^11^ and CrCuFeNi_2_^16^ cause the phase transition from paramagnetic FCC to ferromagnetic BCC structures^11,16^.

Soft magnetic metallic alloys play a prominent role in energy applications^17^. However, conventional soft magnets have their own shortcomings, such as the complicated processing of Fe-Si steels^18^ and the sophisticated procedure involved in the synthesis of nano-crystalline alloys^17^. Nano-crystalline Fe based alloys are used in magnetic applications, such as power transformers, because they exhibit superior soft magnetic properties such as negligible coercivity (H_c_)^19^. However, these alloys are known to behave in a brittle manner after heat treatment^19^.

Recently, we reported the formation of BCC solid solutions in FeMnCoCrAl HEA thin films^20^ by combinatorial synthesis^21^, where phase formation and elastic properties were in good agreement with ab initio predictions and bulk processed alloys^20^. This alloy contains ferromagnetic Fe and Co, as well as Mn which is known to increase the total magnetic moment as observed in Heusler alloys^22^. A wide single BCC stabilizing concentration range of 14 to 26 at.% Al had been identified^20^. In this study, the major emphasis is placed on the Al concentration dependence of BCC phase formation and the resulting magnetic properties. To this end, the Al concentration is varied from 0 to 54 at.% in an effort to determine the Al solubility range in the BCC phase, and to determine the magnetic properties thereof. Hence, the influence of the Al concentration ranging from 3.5 to 13 and 27 to 54 at.% on the phase formation is explored here for the first time. In terms of magnetic properties, the Al concentration range of 0 to 40 at.% is investigated also for the first time. The here adopted research strategy based on combinatorial thin film growth combined with standard as well as nm-scale spatial resolution characterization techniques is shown to be efficient in identifying the complex interplay between composition variation, phase formation and magnetic properties and may serve as a prototype for the efficient identification of composition ranges worthwhile for future in depth investigations.

## Results and Discussions

### Phase formation as a function a chemical composition

**Figure 1:**
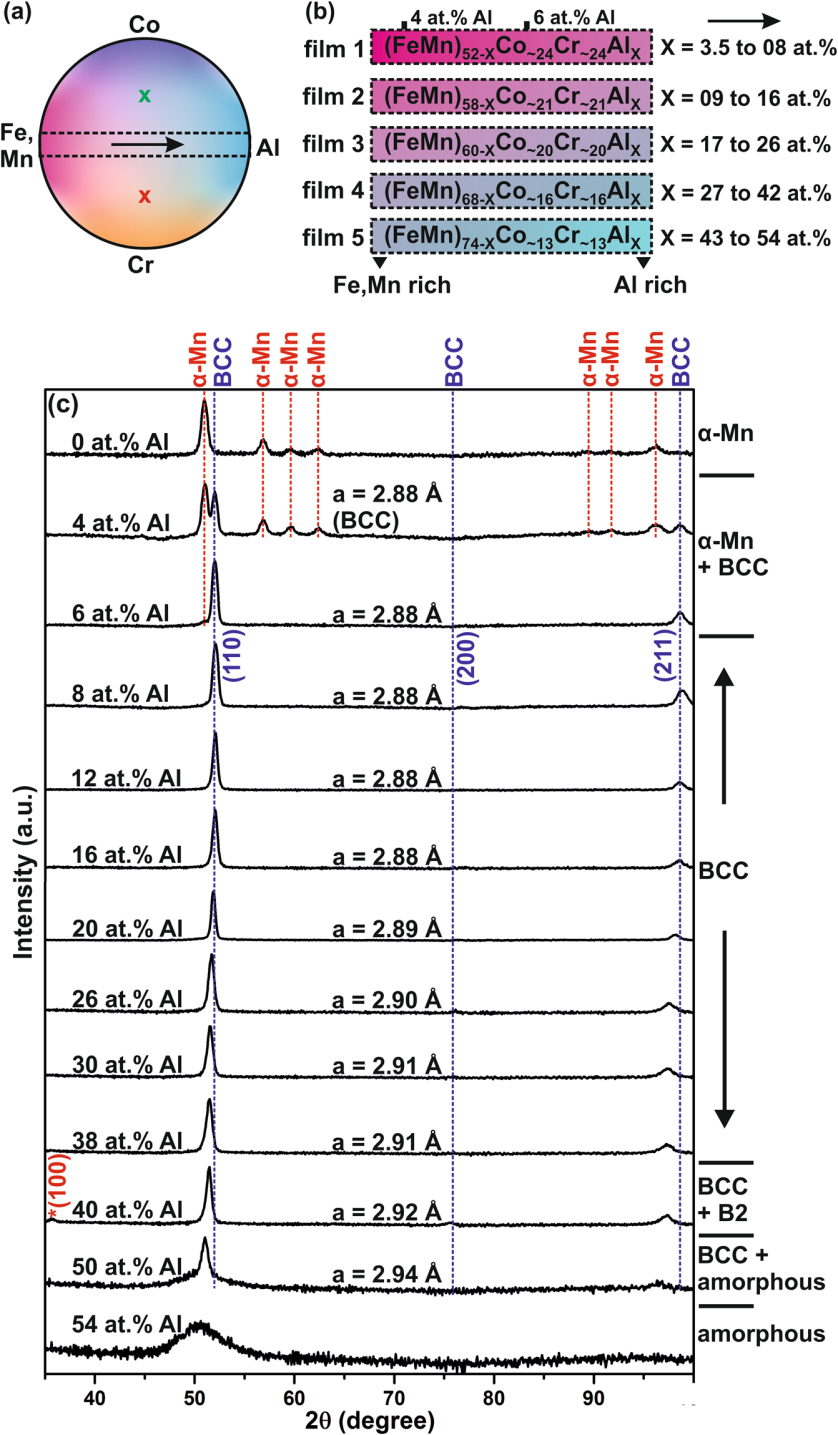
(**a**) Schematic representation of the combinatorial thin film composition spreads (the dotted box indicate the Al gradients and the crosses in the figure corresponds to the characterization regions referred to in Fig. 5), (**b**) Al concentration gradients from five different composition spreads (the markings on film 1 indicate the characterization regions corresponding to 4 and 6 at.% Al) and (**c**) XRD patterns of the as deposited FeMnCoCrAl thin films (the * in the 40 at.% Al diffractogram, is to indicate the (100) B2 reflection).

Five compositionally graded FeMnCoCrAl thin films were deposited, as depicted in Fig. 1(a,b), to study the influence of the Al concentration on phase formation and magnetic properties. The FeMnCoCrAl thin film spreads were characterized by energy dispersive X-ray (EDX) analysis and X-ray diffraction (XRD) regarding chemical composition and phase formation, respectively. The Al concentration was varied from 3.5 ± 0.7 to 54.0 ± 0.7 at.% substituting FeMn. For all as-grown thin film composition spreads the Fe/Mn ratio was 1.0 ± 0.14 and the Co/Cr was 1.0 ± 0.1 along the Al concentration gradient (FeMn → Al, see Fig. 1 (b)). The Co and Cr contents were constant at ~24, 21, 20, 16 and 13 at.% in their respective thin films (1, 2, 3, 4 and 5) along the Al gradient direction as depicted in Fig. 1(b). Composition gradients also exist in the Co to Cr direction, but its impact on phase formation and properties is beyond the scope of this work.

The equiatomic FeMnCoCr (0 at.% Al) composition was compared to the Al containing combinatorial thin films. Structural analysis by XRD shows the phase evolution and lattice parameter variation as a function of Al concentration (Fig. 1(c)). In the upcoming discussions, the FeMnCoCrAl thin films are denoted by the Al concentration in at.% (please refer Fig. 1(b)). The thin film with 0 at.% of Al crystallizes in the α-Mn structure. An additional BCC phase forms upon addition of 4 at.% of Al (the characterization region corresponding to 4 at.% Al on the thin film spread is marked in Fig. 1(b)). The formation of the BCC phase was previously reported for Al concentrations ranging from 14 to 26 at.% in the FeMnCoCr system^20^. The BCC phase becomes more pronounced at the expense of α-Mn phase as the Al concentration is increased to 6 at.%. The thin film with 8 at.% Al, forms a single BCC random solid solution with a lattice parameter of 2.88 Å. Al additions cause an increase in the lattice parameter. For example the film containing 20 at.% of Al exhibits a 0.35% increase in the lattice parameter compared to the film containing 8 at.% of Al. A further increase in Al concentration to 38 at % increases the lattice parameter by 1.04% compared to the film containing 8 at.% of Al. Furthermore, for the 40 at.% Al thin film, an additional superlattice peak corresponding to ordered BCC (B2) is observed at 2θ = 35.7°. Ordering has also been reported upon Al addition to CoCrFeNi and FeCoNiCrMn based bulk HEAs^23–25^, where a sole (100) superlattice reflection^24,25^ in addition to the BCC reflections was observed. However, in the present study, thin films with Al concentrations <40 at.% did not show evidence for the formation of the ordered B2 phase. It should also be noted that the thin film synthesis involves high quenching rates^26^, thus the composition boundaries for phase formation established here might deviate to those obtained by near-equilibrium processing. However, the observed phase formation sequence is of relevance.

As the Al concentration is increased to 50 at.% and 54 at.% the thin films display broader, low intensity peaks, indicating diminishing crystallinity (Fig. 1(c)). This may be due to the increase in the Al content which has a ~15% larger atomic radius than the average radius of the other alloy constituents. The atomic size difference (δ)^27^ of the thin film containing >50 at.% Al is >6.3% calculated according to which was reported to result in the formation of metallic glasses^27^.

**Figure 2:**
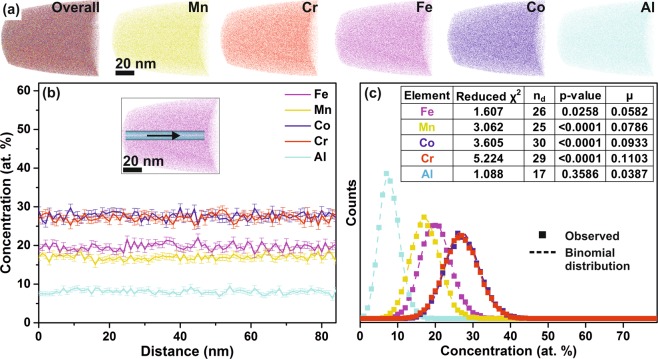
APT (**a**) elemental distribution, (**b**) 1D concentration profile with 1 nm bin width from the 10 nm diameter cylindrical region of interest (shown in inset) and (**c**) frequency distribution analysis (with 100 atoms per bin) of the 8 at.% Al thin film. χ^2^, n_d_, p-value and μ in the inset table designate the deviation of the experimentally observed distribution to the theoretical binomial random distribution. μ as a normalized auto-correlation parameter of χ^2^ can take values between 0 and 1 (where, 0 represents complete randomness and 1 represents clustering).

**Figure 3:**
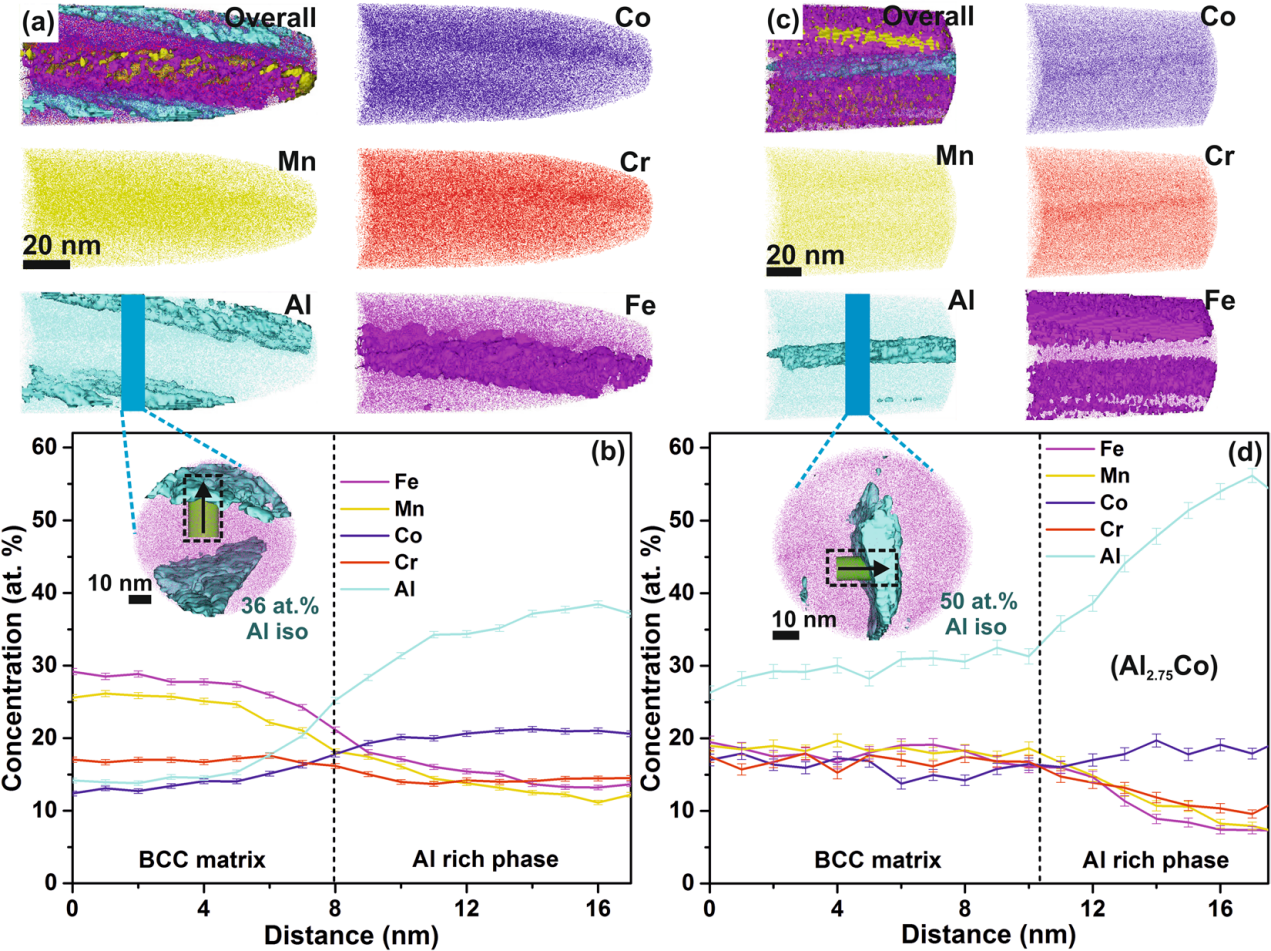
APT elemental distribution (with overlaid iso-concentration surfaces of Fe, Mn and Al) of the (**a**) 26 at.% and (**c**) 40 at.% Al thin film, and 1D concentration profile (with 1 nm bin width) obtained using a 10 nm diameter cylindrical region of interest across the Al rich region of the (**b**) 26 at.% and (**d**) 40 at.% Al thin film (inset in **b and d**: Al iso-concentration surfaces overlaid on Fe distribution).

The local chemical composition of (selected) FeMnCoCrAl thin films is probed by atom probe tomography (APT). Atomic scale elemental distributions of the 8 at.% Al thin film, which crystallizes as a single BCC solid solution (based on XRD), are shown in Fig. 2(a). No evidence of precipitation, elemental segregation or phase separation can be observed. The 1D concentration profiles taken along a 10 nm diameter cylinder displayed in Fig. 2(b) indicates a uniform distribution. In Fig. 2(c) the frequency distribution analysis^28^ of the entire analyzed volume indicates the formation of a random solid solution. Similarly, the equiatomic FeMnCoCrAl (20 at.% Al) thin film exhibits a homogeneous distribution of all the elements, as reported previously^20^. However, for the thin film containing 26 at.% of Al (Fig. 3(a,b)) a nanoscale (anticorrelated) separation is observed between the Al-Co and Fe-Mn-Cr species. The formation of an Al rich region with AlCo/FeMnCr ratio of 1.4 is observed. The APT results of 40 at.% Al HEA, with superlattice peaks at 2θ = 35.7°, corresponding to a B2 ordered phase in XRD (Fig. 1(c)), likewise reveal a clear nano-scale (~6 nm width) separated region (Fig. 3 (c,d)). An Al_2.75_Co based Al rich region exhibiting an AlCo/FeMnCr ratio of 3.0 is observed, which is higher than in the 26 at.% Al thin film with a ratio of 1.4, see Fig. 3(a,b). The enrichment of Al-Co may be accredited to the negative enthalpy of mixing between these constituents. Similar AlCo rich chemical separations with an ordered BCC structure have been reported in a recent transmission electron microscope (TEM) investigation on FeCoCrMnAl single crystals^29^, supporting the notion that the B2 phase corresponds to the Al rich region in high Al containing thin films (Fig. 1 (c)). Even though the FeMnCoCrAl thin films stabilize as a single disordered BCC solid solution at 8 at.% Al (Fig. 1(c)), nanoscale chemical separation (possibly of similar crystal structure and lattice parameter) is observed for 26 at.% of incorporated Al and is not resolved by standard XRD technique (Fig. 1(c)). It is reasonable to assume that the presence of these segregations affect the film properties.

### Magnetic property dependence on chemical composition and microstructure

**Figure 4:**
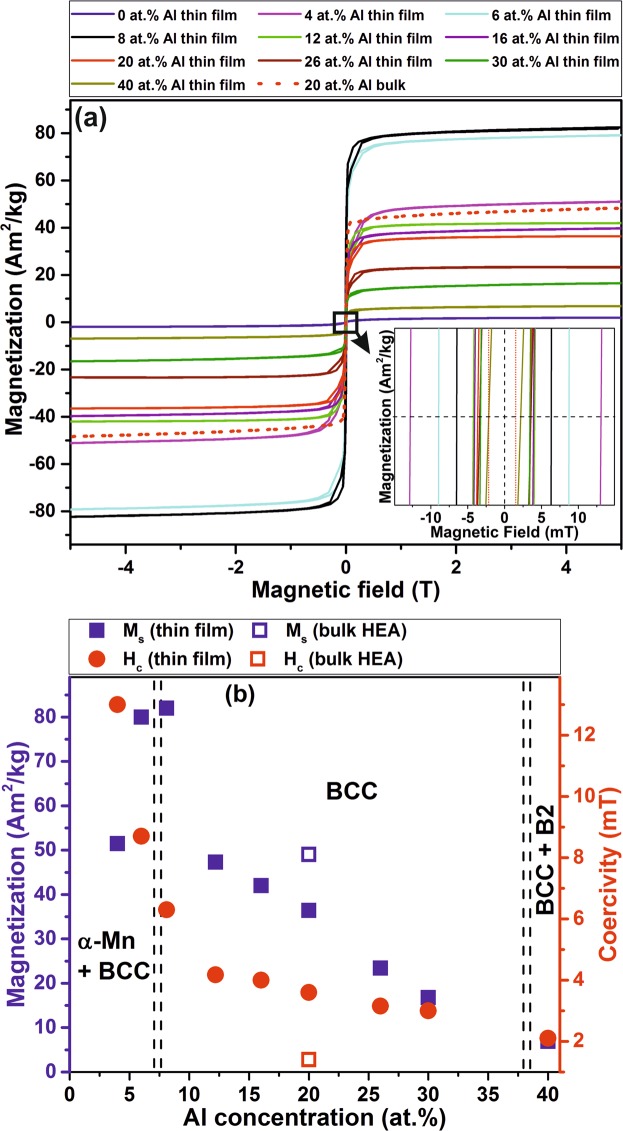
(**a**) Magnetization vs. magnetic field at room temperature for different thin film HEAs, and (**b**) M_s_ and H_c_ variation as a function of Al concentration. The dashed line indicates the composition boundary between multi and single phase thin films.

The room temperature magnetic hysteresis behavior of the FeMnCoCrAl thin films containing various Al concentrations shown in Fig. 4(a). The FeMnCoCr thin film (without Al) is paramagnetic. As discussed above, a BCC phase is formed upon Al addition, see Fig. 1(c), concomitantly a ferromagnetic state is induced.

The here observed magnetic behavior corresponds to a soft ferromagnet with a H_c_ of <15 mT. The M_s_ and H_c_ as a function of Al concentration is displayed in Fig. 4(b). Thin films containing 4 and 6 at.% of Al exhibit in addition to the α-Mn structure also the BCC phase. The increase in M_s_ from 51 to 80 Am^2^/kg is ascribed to the increasing volume fraction of the ferromagnetic BCC phase (see Fig. 1(c)). The largest M_s_ (82 Am^2^/kg) is obtained for the single random BCC solid solution (Fig. 1(c)) containing 8 at.% of Al. A further increase in Al concentration results in a continuous decrease of M_s_, so that the equiatomic BCC thin film (20 at.% Al) possesses a M_s_ of 37 Am^2^/kg, while the 30 at.% Al thin film saturates at 17 Am^2^/kg.

The decrease in M_s_ observed for the BCC HEAs with increasing Al content can be explained, firstly, by considering the magnetic behavior of the constituent elements. Al being paramagnetic, substitutes ferromagnetic species, thus causing a reduction in the number density and hence dilution of ferromagnetic species and consequently a decrease of the overall M_s_. Furthermore, since Al has a ~15% larger atomic radius than the other constituent elements causing >6.3% atomic size difference (δ)^27^, an increase in the Al concentration causes an increase in lattice parameter (as observed in (Fig. 1(c)) and hence, in unit cell volume. It is reasonable to assume that these distortions contribute together with the above discussed dilution of ferromagnetic species by Al to the experimentally observed reduction in overall M_s_.

The thin film containing 40 at.% of Al is weakly ferromagnetic, and exhibits with 7 Am^2^/kg the lowest M_s_. Addition of Al to AlCoCrFeNi^11,25,30^ and FeCoNiCrMnAl^24^ was reported to promote ordering. AlNi enriched phase separation and ordering was reported to decrease the overall moment of the alloy^11^. Al being paramagnetic has a near zero local moment, thereby reduces the ferromagnetic exchange interaction between the ferromagnetic species. Similar Al enriched phase separated regions are observed by the APT investigations of the films containing 26 and 40 at.% of Al (Fig. 3), which is also consistent with the observation of reduced M_s_ compared to the single BCC phase region (Fig. 4(b)).

**Figure 5:**
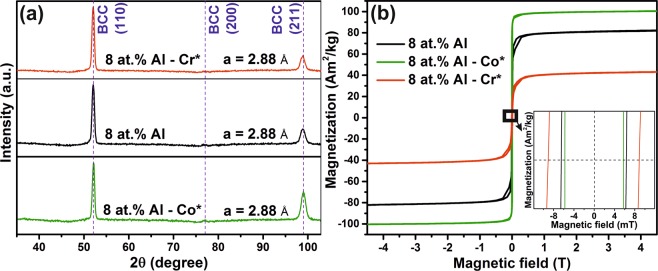
(**a**) XRD patterns and (**b**) room temperature magnetization vs. magnetic field curves of the 8 at.% Al (described in Fig. 1c), 8 at.% Al - Co rich (Co*) and Cr rich (Cr*) thin films.

To ascertain the effect of an increased concentration of Co and Cr on the M_s_, complementary magnetic hysteresis loops were measured for samples containing 8 at % of Al with a composition of ~Fe_22_Mn_22_Co_27_Cr_21_Al_8_ and ~Fe_22_Mn_22_Co_21_Cr_27_Al_8_ (see Fig. 1(a), where the Co and Cr rich regions on the combinatorial thin film schematic are indicated). The phase formation and lattice parameter of the Co and Cr rich regions (Fig. 5(a)) are identical to 8 at.% Al thin film with a Co/Cr ratio of 1.0 ± 0.1 (Fig. 1(c)), whereas a considerable difference is observed for the M_s_ (Fig. 5(b)). The Co rich thin film is characterized by a 21% increased M_s_ (99 Am^2^/kg), while the thin film enriched with antiferromagnetic Cr possesses 46% smaller M_s_ (44 Am^2^/kg) than the 8 at.% Al thin film with a Co/Cr ratio of 1.0 ± 0.1, which saturates at 82 Am^2^/kg.

**Figure 6:**
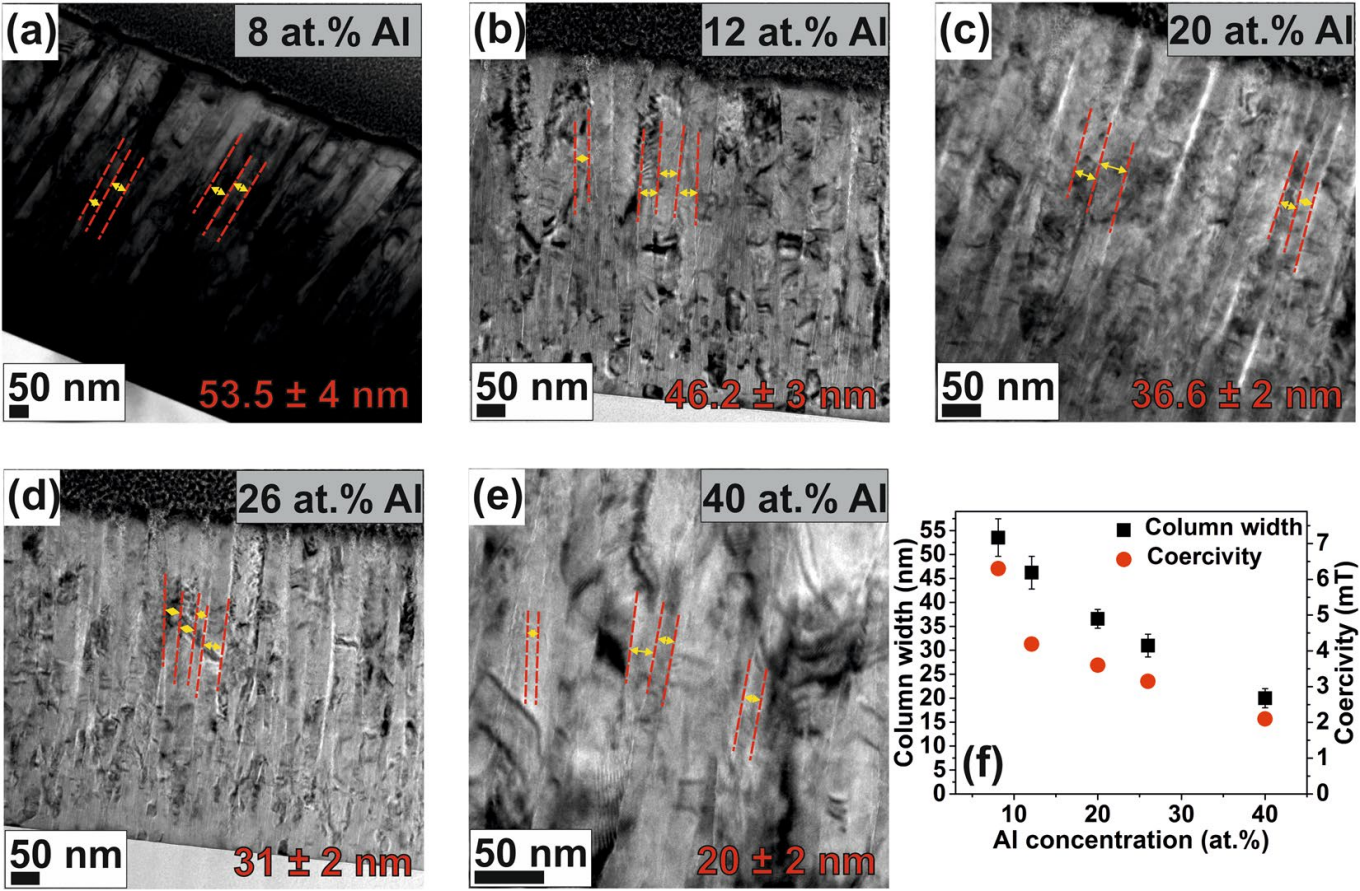
(**a**–**e**) TEM bright-field images showing columnar grain morphology (line markings overlaid to indicate the column width), and (**f**) column width dependence of the room temperature H_c_ for FeMnCoCrAl thin films with varying Al concentration.

A low H_c_ value is another prerequisite for soft magnets and the H_c_ of soft magnets is strongly dependent on the grain size^17^ and the defect structure. The 4 at.% Al thin film with both paramagnetic α-Mn and ferromagnetic BCC phase (see Figs 1(c) and 4(b)) exhibits the highest Hc of 13 mT. The FeMnCoCrAl thin films exhibit columnar grain morphologies (Fig. 6), and the average column width of the thin films as a function of Al concentration is correlated with the measured coercivities. TEM bright field images of the thin film cross section are presented in Fig. 6(a–e). Figure 6(f) shows the column width of various thin films as a function of the Al concentration. As the Al concentration is increased, the column width is decreased. The 8 at.% Al thin film has a column width of ~54 nm, while the 40 at.% Al thin film exhibited the smallest column width of ~20 nm. The corresponding coercivities of the 8 and 40 at.% Al HEAs were 6.3 and 2.1 mT, respectively. The Al concentration induced decrease in the column width of 63% results in a 67% decrease in the coercivity. It should be noted that the coercivity change in the present study is a consequence of both composition and grain size variation. More detailed investigations on factors affecting H_c_ and the effects of magnetic anisotropy are subject of future work.

The magnetization of FeMnCoCrAl equiatomic bulk HEA crystallizing according to XRD in the BCC structure^20^, is compared to the near equiatomic FeMnCoCrAl thin film in Fig. 4. While the FeMnCoCr bulk HEA without Al additions is paramagnetic, the BCC bulk FeMnCoCrAl HEA^20^ is (soft) ferromagnetic (Fig. 4(a), dotted curve). The M_s_ and H_c_ of the bulk HEA are 49 Am^2^/kg and 1.4 mT, respectively (Fig. 4(b)), while the equiatomic FeMnCoCrAl thin film saturated at 37 Am^2^/kg with a H_c_ of 3.5 mT. The M_s_ of the thin film is 24% smaller than its bulk counterpart. This difference may at least in part be attributed to the magnetic anisotropy of textured thin films with columnar morphology^31^. On the other hand, the coercivity of the thin film is 2.5 times larger than the bulk, most likely due to the different microstructures. The bulk HEAs exhibit equiaxed, µm sized grains^20^, while the thin film morphology is clearly columnar.

### Electronic structure of FeMnCoCrAl HEA

**Figure 7:**
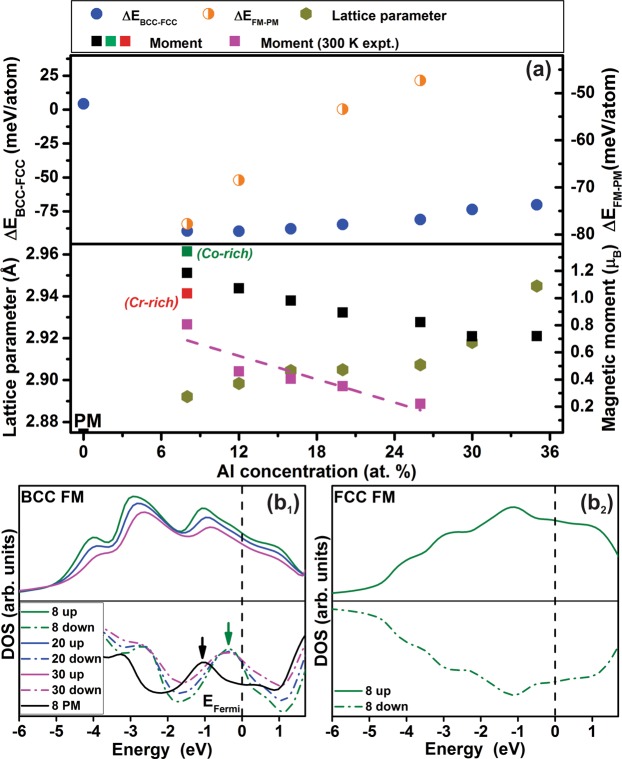
DFT calculations: (**a**) Internal energy difference (ΔE), lattice parameter and total magnetic moment of BCC HEAs with varying Al concentrations, and (**b**_1,2_) spin polarized total DOS for 8 at.% Al HEA in BCC and FCC structure. The Fermi level is set to 0 eV. The arrows in (**b**_1_) indicate the pseudogap position of BCC FM (green) with respect to the BCC PM (black) configuration.

Ab initio density functional theory (DFT) calculations were performed to investigate the influence of the Al concentration on the phase stability of BCC and FCC phases as well as the magnetic behavior. The magnetic moments were determined within the Exact muffin tin orbitals (EMTO) formalism (as described in the methods section) and the variation of total magnetic moment with varying Al concentration is compared with previously reported internal energy difference (ΔE_BCC-FCC_) data between the BCC and FCC structures^20^, as displayed in Fig. 7(a).

The phase stability data (ΔE_BCC-FCC_) for the FeMnCoCrAl system were calculated only for the BCC and FCC structures, as these are the crystal structures observed in 3d transition metal based HEAs^27^. The FeMnCoCr configuration is stable in the paramagnetic (PM) FCC structure, whereas for the Al containing FeMnCoCrAl configurations, the ferromagnetic (FM) BCC structure (Fig. 7(a)) is the stable configuration, and in good agreement with the here presented experimental results (Fig. 1(c)). The BCC phase stability exhibits a slightly decreasing trend when the Al content is increased.

The total magnetic moment of the BCC FeMnCoCrAl configuration is shown in Fig. 7(a). Regarding the local moment of the individual elements, Fe, Mn and Co possess positive, Cr exhibits negative (antiparallel) and Al has near zero magnetic moment, as in their ground states. The calculated magnetic moment (for the experimentally obtained single phase region) is highest for the BCC configuration containing 8 at.% Al. Further Al addition to the system (>8 at.%) leads to a decrease in the magnetic moment, and an increase in the unit cell volume (as reflected from the calculated lattice parameter results in Fig. 7(a)), likely diluting and weakening the ferromagnetic exchange interactions. As expected, the magnitude of the calculated 0 K DFT total magnetic moment values are higher than that of the 300 K experimental data^32^. Nevertheless, the trend of the calculated moment vs Al concentration is consistent with the experimental data. Magnetic moments were also calculated for the 8 at.% Al content of the Co and Cr rich compositions described in the previous section (see Fig. 5). Also, the trend in total moment values for both Cr rich and Co rich compositions are consistent with the experimental observations: An increase in antiferromagnetic Cr content decreases the overall magnetic moment, while Co additions result in an increase compared to the 8 at.% Al thin film with a Cr/Co ratio of 1.0 ± 0.1. To elucidate the role of electronic structure on ferromagnetism, spin polarized density of states (DOS) data are presented in Fig. 7(b_1,2_). The total density of states was evaluated for two different spin polarized equilibrium configurations, namely (i) the BCC and (ii) the FCC state. The electronic structure analysis of the random BCC configuration containing 8 at.% of Al in terms of up and down spin states is shown in Fig. 7(b_1,2_). The distribution of both up and down spin states is clearly asymmetric (Fig. 7(b_1_)), indicating ferromagnetism. Conversely, the FCC structure displays a symmetric up and down spin states (Fig. 7(b_2_)), suggesting magnetization cancellation. Furthermore, ferromagnetism in the BCC configuration decreases the number of states by pseudogap formation at the Fermi level with respect to the paramagnetic state, as indicated by green and black arrows in Fig. 7(b_1_), and thereby stabilizes the system. This argumentation is based on the notion that if a Fermi level is located in a pseudogap, separating bonding and antibonding states, the alloy is stabilized^33^.

### Curie temperature and electrical resistivity of FeMnCoCrAl thin films

**Figure 8:**
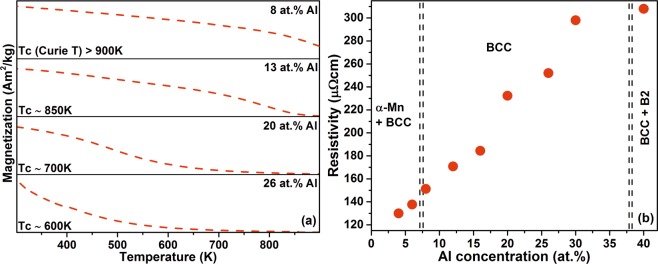
(**a**) Temperature dependence of magnetization at 10 mT applied field, and (**b**) room temperature electrical resistivities of selected thin films with varying Al concentrations. The dashed line indicates the composition boundary between multi and single phase thin films.

Magnetic transitions determine the operating temperature ranges of ferromagnets. The temperature dependence of magnetization for the thin films is shown in Fig. 8(a). The 8 at.% Al single BCC phase (Fig. 1(c)) thin film possesses the highest T_c_ > 900 K (which is beyond our measurement limit), making these alloys viable candidates for applications of soft magnetic materials at high operation temperatures.

A decreasing trend is observed in T_c_ with an increase in the Al concentration, where the 12 at.% Al and equiatomic 20 at.% Al thin film showed a T_c_ of ~850 K and of 700 K, respectively, while the high Al containing 26 at.% Al thin film exhibits the lowest magnetic transition temperature at ~600 K. The observed decrease in T_c_ with increasing Al content can be rationalized based on the internal energy difference between the ferromagnetic and paramagnetic BCC FeMnCoCrAl configurations. The magnetic transition from ferromagnetic to paramagnetic state occurs at the T_c_ and the energy difference (Fig. 7(a), ΔE_FM-PM_) between the two states gives an indication about the transition temperature. The energy difference is maximized for the 8 at.% Al alloy with a highest T_c_ and decreases with increasing Al content. The 26 at.% Al HEA exhibits the minimum energy difference and hence a lower T_c_. The observed inverse relationship between T_c_ and the Al concentration can also be rationalized by considering the paramagnetic nature of Al. We have demonstrated that the Al concentration induced reduction in T_c_ without compromising phase stability may enable application of this material system in magneto-caloric devices where Curie temperatures near room temperature are required, as well as in applications requiring high Curie temperatures like power transformers and high frequency inductors.

Large electrical resistivity is another desired property of soft magnetic materials to minimize eddy current losses^14,17^. The sheet resistivity of the thin films with varying Al concentration was measured at room temperature (Fig. 8(b)). The thin film HEAs exhibit large resistivity values (>130 µΩ cm), when compared to conventional soft magnets^17^. The single phase BCC films containing 8 at.% of Al exhibits a resistivity of 151 µΩ cm, and the resistivity increases as the Al concentration is increased. The addition of large atomic radius Al to the system causes large lattice distortions (as seen in the XRD results) in addition to the inherent topological distortion and chemical randomness in the HEAs^14^. These distortions were reported to cause more electron scattering and hence higher electrical resistivities are observed^14^. From microstructure point of view, the grain size and fraction of grain boundaries also influence the electrical resistivity. As the concentration of Al increases the nano-column width (Fig. 6) of the FeMnCoCrAl thin films decreases, thereby increasing the fraction of grain boundaries causing a concomitant increase in the sheet resistivities of the thin films. Furthermore, in terms of electronic structure the configurations containing 8 at.% of Al exhibit a larger number of states (Fig. 7(b_1_)) at the Fermi level, rendering the materials with a lower intrinsic resistivity than configurations with larger Al concentrations.

## Conclusions

The effect of Al content (3.5–54 at.%) on phase formation and magnetic properties of combinatorially deposited FeMnCoCrAl thin films has been investigated systematically by using conventional and spatially resolved characterization techniques as well as DFT. Al additions to the FeMnCoCr crystallizing in the α-Mn structure cause the formation of a BCC phase. Thin films containing 8 at.% of Al exhibit a single phase BCC structure and atom probe tomography revealed that the solid solution identified by XRD is in fact disordered. However, thin films containing 26 at.% of Al contain only the BCC phase as identified using XRD showed clear evidence for the presence of Al-Co rich (~10 nm) nano-scale segregations by atom probe tomography. Similar segregations were also identified for the sample containing 40 at.% of Al which exhibited the presence of B2 type ordering by XRD. DFT predictions on the effect of Al additions to FeMnCoCr causing the formation of the BCC phase are consistent with the experimental data. Al additions cause the formation of a pseudogap at the Fermi level rendering the ferromagnetic BCC phase more stable than paramagnetic FCC phase. Magnetometry measurements reveal a paramagnetic behavior in the FeMnCoCr system crystallizing in the α-Mn structure. Upon Al addition the BCC structure is formed, exhibiting a (soft) ferromagnetic behavior, where the thin film containing 8 at.% of Al displays with 82 Am^2^/kg the maximum magnetization. Further increase in the non-ferromagnetic Al content beyond 8 at.% decreases the overall M_s_. This can be understood by considering the substitution of ferromagnetic species by paramagnetic Al as well as by the concomitantly induced lattice distortions. The measured trend of the Al concentration induced reduction in magnetization is consistent with DFT predictions. The here employed experimental and theoretical research strategy was shown to be efficient in explaining the Al concentration dependent phase formation and magnetic properties, and shows therefore great potential for future design of efficient soft magnetic HEA thin films.

## Methods

### Experimental details

Combinatorial FeMnCoCrAl HEA thin films, exhibiting Al concentration gradients were grown on single crystalline Si (100) and Al_2_O_3_ (0001) (for temperature dependent magnetization studies) substrates. The phase formation behavior was similar on both the substrates. An equiatomic FeMn target (with ~99.95% purity), as well as elemental Co, Cr and Al targets (with ~99.95% purity) were employed in a co-sputtering geometry^20,34^ (Fig. 1(a)). A constant power density was applied to the FeMn (7.5 W cm^−2^) and Co, Cr (~4 W cm^−2^ each) cathodes, while for the Al target, the power density was varied from 2 to 7.5 W cm^−2^ to obtain multiple Al concentration gradients (Fig. 1(b)), which facilitate the efficient investigation of the effect of Al content on phase formation and magnetic properties. In addition, a four component FeMnCoCr thin film was also deposited to study the phase formation in the absence of Al. The base pressure of the system was 2 × 10^−5^ Pa and Ar (0.4 Pa) gas was used as a sputtering gas. No intentional heating was applied to the substrates during deposition. The deposition was carried out for 22 min, which resulted in a film thickness of ~1 µm. The deposition geometry and other process specifics of the combinatorial setup are described elsewhere^34^. The substrate temperature before venting was <80 °C to minimize the effect of chemical reactions of the film surface with the atmosphere^35^.

The chemical composition of the thin film spreads were quantified using EDX analysis, in a JEOL-JSM-6480 scanning electron microscope (SEM) at an acceleration voltage of 15 kV. The phase formation of the thin film composition spreads was determined using XRD, employing a Bruker D8 general area detection diffraction system in grazing incidence geometry (Ω = 10°) with Co Kα radiation.

The microstructure of the samples were characterized using a FEI Tecnai F20 TEM^36^ operated at 200 kV. Near atomic scale elemental distribution of the FeMnCoCrAl thin films were investigated at 60 K tip temperature by APT. A Cameca Local Electrode Atom Probe (LEAP) 4000X HR instrument, operated in laser pulse mode, employing a 30 pJ pulse energy at a constant applied frequency of 250 kHz was used. Post measurement data reconstruction and analysis were done using the IVAS 3.6.10a software provided by Cameca Instruments. Samples for both TEM and APT measurements from the thin film spreads were prepared by Ga ion beam milling in a dual beam FEI Helios NanoLab 660 microscope.

The magnetization behavior of the FeMnCoCrAl HEA thin films at various magnetic fields and temperatures were probed using the Vibrating Sample Magnetometer (VSM) option at a Physical Property Measurement System (PPMS^®^) from Quantum Design. A maximum field of 5 T was applied to saturate the HEA thin films, while the temperature dependent magnetization was studied at a constant applied field of 10 mT. Room temperature electrical resistance measurements of the combinatorial thin film library were carried out using a custom built four point probe setup. Current and potential difference measurements were performed using a Keithley digital multimeter (2611 B) to determine the specific sheet resistivity of the FeMnCoCrAl thin films using the Van-der-Pauw method^37^.

### Ab initio calculations

Ab initio DFT calculations on the FeMnCoCrAl based system for various Al concentrations were performed to determine the phase stability, magnetic moments of the corresponding stable states and the spin polarized DOS. EMTO, based on Green’s functions and full charge density, as described in^20,38,39^ were utilized. The generalized gradient approximation was applied for the density functionals and ion cores were kept frozen. The integration in the Brillouin zone was carried out on a 13 × 13 × 13 k-mesh and the total energy convergence criterion was set to 10^−7^ Ry. The compositional disorder for HEA was described within the coherent potential approximation (CPA). The CPA derived data have been proven to be consistent with experimental data for multicomponent HEAs^40,41^. The magnetic state of both the BCC and FCC HEA was treated in two ways, the magnetically ordered configurations with ferromagnetic polarization and the magnetically disordered configurations with CPA randomized magnetic moments, which is known as the disordered local moment (DLM) model^42^. This DLM model provides a reasonable approximation of the paramagnetic state above the transition temperature^20,43^. The local magnetic moment of the HEAs was evaluated based on generalized gradient approximation by Perdew-Burke-Ernzerhof^44^.

## Data Availability

The data and samples analyzed during the current study are available from the corresponding author on reasonable request.
